# Metatranscriptomic Sequencing Reveals Host Species as an Important Factor Shaping the Mosquito Virome

**DOI:** 10.1128/spectrum.04655-22

**Published:** 2023-02-14

**Authors:** Cixiu Li, Shuqi Liu, Hong Zhou, Wei Zhu, Mingxue Cui, Juan Li, Jiao Wang, Jiangyun Liu, Jin Zhu, Weiping Li, Yuhai Bi, Michael J. Carr, Edward C. Holmes, Weifeng Shi

**Affiliations:** a Department of Pathogen Biology, School of Clinical and Basic Medicine, Shandong First Medical University & Shandong Academy of Medical Sciences, Ji’nan, China; b Key Laboratory of Emerging Infectious Diseases in Universities of Shandong, Shandong First Medical University & Shandong Academy of Medical Sciences, Taian, China; c Mengla County Center for Disease Control and Prevention, Mengla, China; d Xishuangbanna Prefecture Center for Disease Control and Prevention, Jinghong, China; e Key Laboratory of Pathogenic Microbiology and Immunology, Institute of Microbiology, Chinese Academy of Sciences, Beijing, China; f National Virus Reference Laboratory, School of Medicine, University College Dublin, Dublin, Ireland; g International Collaboration Unit, International Institute for Zoonosis Control, Hokkaido University, Sapporo, Japan; h Sydney Institute for Infectious Diseases, School of Medical Sciences, The University of Sydney, Sydney, New South Wales, Australia; i School of Public Health, Shandong First Medical University & Shandong Academy of Medical Sciences, Ji’nan, China; Changchun Veterinary Research Institute

**Keywords:** mosquito, virome, evolution, ecology, metatranscriptomics

## Abstract

Mosquitoes are important vector hosts for numerous viral pathogens and harbor a large number of mosquito-specific viruses as well as human-infecting viruses. Previous studies have mainly focused on the discovery of mosquito viruses, and our understanding of major ecological factors associated with virome structure in mosquitoes remains limited. We utilized metatranscriptomic sequencing to characterize the viromes of five mosquito species sampled across eight locations in Yunnan Province, China. This revealed the presence of 52 viral species, of which 19 were novel, belonging to 15 viral families/clades. Of particular note was Culex hepacivirus 1, clustering within the avian clade of hepaciviruses. Notably, both the viromic diversity and abundance of *Aedes* genus mosquitoes were significantly higher than those of the *Culex* genus, while Aedes albopictus mosquitoes harbored a higher diversity than Aedes aegypti mosquitoes. Our findings thus point to discernible differences in viromic structure between mosquito genera and even between mosquito species within the same genus. Importantly, such differences were not attributable to differences in sampling between geographical location. Our study also revealed the ubiquitous presence of the endosymbiont bacterium *Wolbachia*, with the genetic diversity and abundance also varying between mosquito species. In conclusion, our results suggested that the mosquito host species play an important role in shaping the virome’s structure.

**IMPORTANCE** This study revealed the huge capability of mosquitoes in harboring a rich diversity of RNA viruses, although relevant studies have characterized the intensively unparalleled diversity of RNA viruses previously. Furthermore, our findings showed discernible differences not only in viromic structure between mosquito genera and even between mosquito species within the same genus but also in the genetic diversity and abundance of *Wolbachia* between different mosquito populations. These findings emphasize the importance of host genetic background in shaping the virome composition of mosquitoes.

## INTRODUCTION

Numerous mosquito-borne viruses are able to cause disease in humans and other vertebrates, with the potential for major epidemics and pandemics that disrupt global public health and threaten human and animal populations (1, 2). With the deployment of next-generation sequencing, particularly metatranscriptomics (total RNA sequencing), an enormous genetic diversity of viruses in mosquitoes has been identified (3, 4), the majority of which are insect specific (5, 6) and are distinct from viruses of medical importance. As such, the presence of disease-causing viruses in mosquitoes is probably the exception rather than the rule. However, how ecological factors impact the composition and diversity of mosquito viromes remains unclear (3, 7), limiting our understanding of the ecological drivers of host-switching and spillover events and their public health risks (8). In addition, the pathogenicity of most mosquito-borne viruses is poorly understood, even though some may pose a risk to public health or modulate the transmission of pathogenic viruses (9).

Jinghong City, part of the Xishuangbanna Dai Autonomous Prefecture of Yunnan Province, is located on the southern border of China and geographically adjacent to Laos, Myanmar, and Vietnam, where some tropical viral infectious diseases are endemic (10, 11). In recent years, mosquito-associated infectious diseases, such as dengue fever and Japanese encephalitis (12, 13), have been imported into Xishuangbanna. In addition, the stable tropical climate in Xishuangbanna facilitates mosquito breeding and production (10), constituting an important driver of infectious disease outbreaks. Indeed, several large-scale dengue outbreaks have been reported in Xishuangbanna since the first documented epidemic there in 2013 (12, 14, 15). Therefore, characterization of the virus spectrum in the key mosquito species (particularly species from the *Aedes* and *Culex* genera that are known to harbor pathogenic viruses responsible for epidemics in human and animal populations) and identification of possible associations between mosquito species and virome structures are of vital importance to prevent and control future tropical disease outbreaks here and potentially elsewhere.

Herein, we characterized the total transcriptomes of 56 mosquito pools, comprising 991 mosquitoes from five invertebrate species collected from eight locations in Jinghong, Xishuangbanna. We analyzed the genetic diversity of RNA viruses in these mosquito species and identified the complete coding sequences of 52 RNA viruses, including 19 previously undescribed viruses. We further determined the evolutionary relationships of the novel viruses identified here and revealed an association between mosquito vector species and virome structure by comparing the compositions and structures of the viral communities within different hosts.

## RESULTS

### The mosquito viromes.

**FIG 1:**
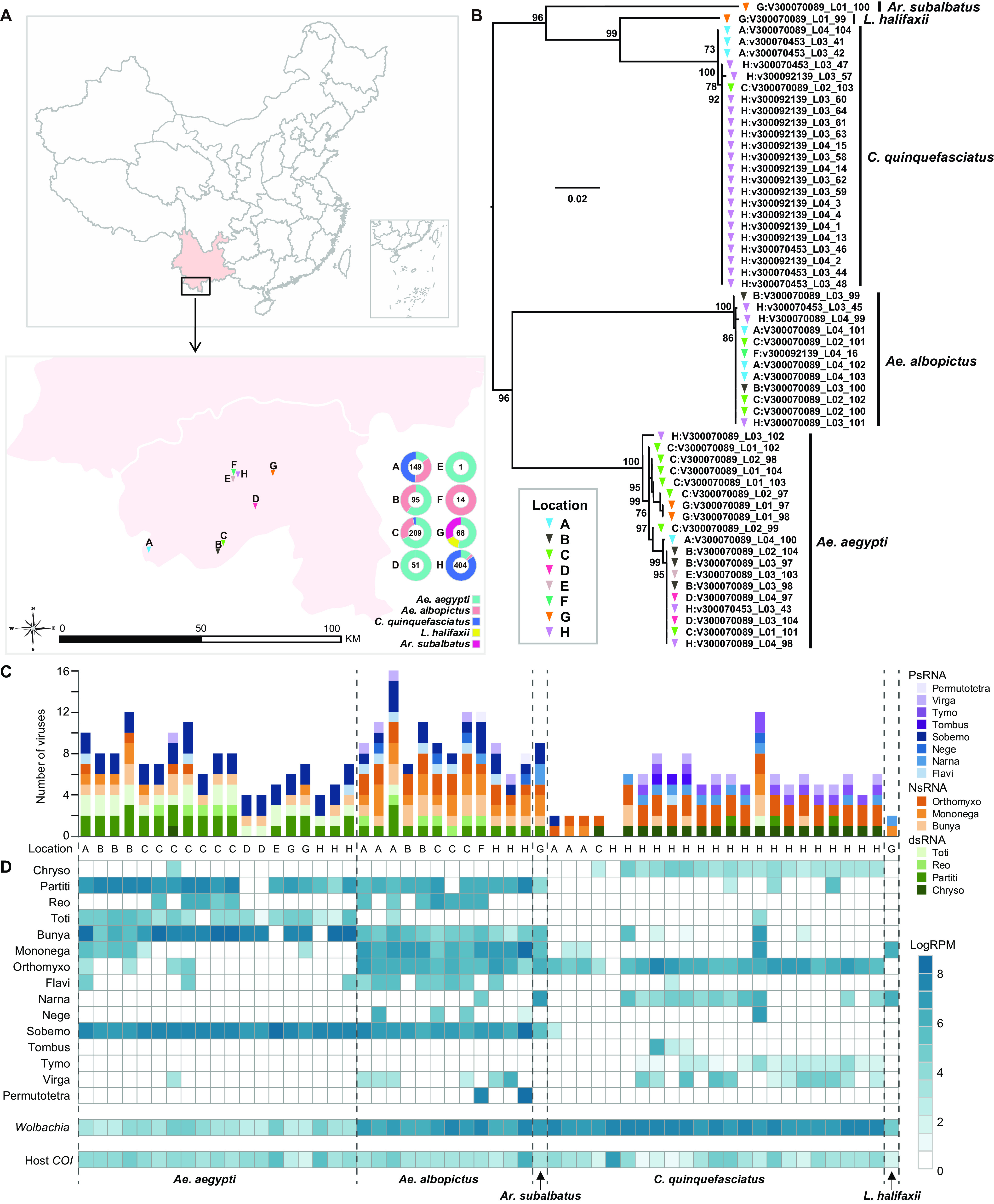
Geographic locations and the species of the mosquito samples and overview of the RNA viruses and the *Wolbachia* bacteria identified in this study. (A) Sampling sites in Jinghong City and the mosquito species composition of each site. The mosquito species and the number of mosquitoes are shown in different colors. The maps were first created using ArcMap v10.4.1 and further edited using Adobe Illustrator 2020. (B) Species identification was based on phylogenetic analysis of the cytochrome *c* oxidase (*COI*) gene of the mosquitoes. (C) Number of viral species identified in each library, colored by virus type; (D) heat map showing the abundance (measured by RPM) of microbial species and the host *COI* gene in each of the 56 pools sequenced here.

A total of 991 mosquitoes were collected in 2018 from eight locations (A to H) in Jinghong City, Yunnan Province, China (Fig. 1A; see Table S1 in the supplemental material). The mosquitoes comprised five species: Culex quinquefasciatus (*n* = 425), Aedes aegypti (*n* = 355), Aedes albopictus (*n* = 179), Lutzia halifaxii (*n* = 10), and Armigeres subalbatus (*n* = 22) (Fig. 1B; Table S1). Samples were pooled into 56 libraries based on mosquito species and collection location (Table S1). Metatranscriptomic sequencing generated between 34,777,328 and 192,424,498 reads per library (Table S1).

Subsequent analyses revealed the complete coding sequences of 52 RNA viruses, 19 of which were novel viruses due to a sequence similarity of <90% in the RNA-dependent RNA polymerase (RdRp) protein (Table 1). All viruses fell within known viral families or orders: *Chrysoviridae* (*n* = 1), *Partitiviridae* (*n* = 5), *Reoviridae* (*n* = 1), *Totiviridae* (*n* = 3), *Bunyavirales* (*n* = 7), *Mononegavirales* (*n* = 9), *Orthomyxoviridae* (*n* = 6), *Flaviviridae* (*n* = 3), *Narnaviridae* (*n* = 3), *Negevirus* (*n* = 2), *Solemoviridae* (*n* = 6), *Tombusviridae* (*n* = 1), *Tymoviridae* (*n* = 2), *Virgaviridae* (*n* = 2), and *Permutotetraviridae* (*n* = 1) (Table 1).

**TABLE 1:** Mosquito RNA viruses discovered in this study

Virus no.	Classification	Virus name^*a*^	Segment no.	Length (nt)	Closest relative	RdRp aa identity (%)	RPM^*b*^
dsRNA viruses							
1	*Chrysoviridae* related	Shuangao chryso-like virus 1 YN2018	4		Shuangao chryso-like virus 1 strain mosWSB68555	100	140
2	*Partitiviridae* related	Aedes partiti-like virus 1 YN2018	2		Aedes partiti-like virus 1 isolate Jane	100	2,539
3	*Partitiviridae* related	Aedes partiti-like virus 2*	1	1,709	Culex tritaeniorhynchus partitivirus 17CxITNGK-Ctr	61.17	1,770
4	*Partitiviridae* related	Armigeres partiti-like virus 1*	1	1,486	Aedes partiti-like virus 1 isolate Jane	83.47	57
5	*Partitiviridae* related	Sonnbo virus YN2018	3		Sonnbo virus isolate OTU64	99.46	179
6	*Partitiviridae* related	Verdadero virus YN2018	2		Verdadero virus isolate Vv.PozaRica20	99.62	663
7	*Reoviridae* related	Aedes reo-like virus 1*	2	4,216/3,096	Shenzhen reo-like virus 2	30.00	922
8	*Totiviridae* related	Aedes aegypti toti-like virus YN2018	1		Aedes aegypti toti-like virus strain 2017-PB-AAM-5	98.70	32
9	*Totiviridae* related	Aedes aegypti totivirus YN2018	1		Aedes aegypti totivirus GH115	97.16	289
10	*Totiviridae* related	Culex toti-like virus 1*	1	7,194	Fitzroy Crossing toti-like virus 2 isolate FCToLV2/pool-7	73.20	81
−ssRNA viruses							
11	*Bunyavirales*	Aedes bunya-like virus 1*	1	7,731	Salarivirus Mos8CM0	48.54	196
12	*Bunyavirales*	Barstukas virus YN2018	3		Barstukas virus isolate CMS002_026c_WVAL	99.54	146
13	*Bunyavirales*	Culex bunyavirus 1 YN2018	1		Bunyavirales sp. strain YX392	96.06	683
14	*Bunyavirales*	Culex quinquefasciatus bunyavirus 1*	3	7,102/3,676/1,642	Qingnian mosquito virus strain YC179	88.78	271
15	*Bunyavirales*	Culex phasma-like virus YN2018	3		Culex phasma-like virus strain mos191gb30698	99.91	627
16	*Bunyavirales*	Phasi Charoen-like phasivirus YN2018	3		Phasi Charoen-like phasivirus strain Zhanjiang01	99.28	4,271
17	*Bunyavirales*	Zhee mosquito virus YN2018	1		Zhee mosquito virus strain XC1-8	99.22	196
18	*Mononegavirales*	Aedes rhabdovirus 1*	1	10,320	Merida virus isolate CC_H	48.08	21
19	*Mononegavirales*	Aedes albopictus anphevirus YN2018	1		Aedes albopictus anphevirus strain USA	99.46	5,462
20	*Mononegavirales*	Aedes anphevirus YN2018	1		Aedes anphevirus isolate Nakhon Nayok	99.71	344
21	*Mononegavirales*	Armigeres rhabdo-like virus 2*	1	10,143	San Gabriel mononegavirus isolate San Gabriel Valley	52.71	37
22	*Mononegavirales*	Armigeres rhabdo-like virus 1*	1	12,152	Formosus virus isolate U30	72.52	169
23	*Mononegavirales*	Culex rhabdo-like virus 2*	1	11,680	Culex rhabdo-like virus strain CRVL/Los Angeles	73.38	637
24	*Mononegavirales*	Culex mononega-like virus 2 YN2018	1		Culex mononega-like virus 2 strain mos191X56446	99.76	26
25	*Mononegavirales*	Culex quinquefasciatus rhabdo-like virus 1	1	11,508	Culex pseudovishnui rhabdo-like virus 17NGK-Cps-217	94.67	1,149
26	*Mononegavirales*	San Gabriel mononegavirus YN2018	1		San Gabriel mononegavirus isolate San Gabriel Valley	98.67	93
27	*Orthomyxoviridae* related	Aedes orthomyxo-like virus 2 YN2018	4		Aedes orthomyxo-like virus 2 isolate Italy Rome	99.35	409
28	*Orthomyxoviridae* related	Armigeres orthomyxo-like virus 1*	5	2,437/2,425/2,218/1,835/1,509	Usinis virus isolate CMS002_026e_WVAL	69.27	437
29	*Orthomyxoviridae* related	Wuhan mosquito virus 6 YN2018	6		Wuhan mosquito virus 6 strain YX592	100	1,659
30	*Orthomyxoviridae* related	Guadeloupe mosquito quaranja-like virus 1 YN2018	7		Guadeloupe mosquito quaranja-like virus 1 isolate CMS002_017a_SAND	99.35	25
31	*Orthomyxoviridae* related	Usinis virus YN2018	8		Usinis virus isolate CMS002_026e_WVAL	99.62	820
32	*Orthomyxoviridae* related	Wuhan mosquito virus 4 YN2018	5		Wuhan mosquito virus 4 strain XC3-4	100	565
+ssRNA viruses							
33	*Flaviviridae* related	Aedes flavivirus YN2018	1		Aedes flavivirus strain Bangkok	99.67	287
34	*Flaviviridae* related	Cell fusing agent virus YN2018	1		Cell fusing agent virus isolate KPP	99.19	107
35	*Flaviviridae* related	Culex hepacivirus 1*	1	8,997	Jogalong virus isolate P1-10	54.38	11
36	*Narnaviridae* related	Zhejiang mosquito virus 3 YN2018	1		Zhejiang mosquito virus 3 strain mosHB236413	95.23	485
37	*Narnaviridae* related	Hubei mosquito virus 3 YN2018	1		Hubei mosquito virus 3 strain 3mos6141	98.2	806
38	*Narnaviridae* related	Mosquito narna-like virus 1*	1	3,160	Ochlerotatus-associated narna-like virus 2 isolate acc_9.11s	36.17	114
39	*Negevirus* related	Aedes albopictus negev-like virus YN2018	1		Aedes albopictus negev-like virus isolate Aag2.wAlbB	99.12	399
40	*Negevirus* related	Culex quinquefasciatus negev-like virus 1*	1	10,894	Parry's Creek negev-like virus 1 isolate PCNLV1/pool-12	90.05	1,132
41	*Solemoviridae* related	Aedes sobemo-like virus 1*	1	2,782	Hubei sobemo-like virus 41 strain spider133517	87.6	11
42	*Solemoviridae* related	Guadeloupe mosquito virus YN2018	2		Guadeloupe mosquito virus isolate CMS002_019a_SAND	99.55	3,013
43	*Solemoviridae* related	Guangzhou sobemo-like virus YN2018	2		Guangzhou sobemo-like virus strain 18-GZ-adult	99.77	5,457
44	*Solemoviridae* related	Hubei sobemo-like virus 41 YN2018	1		Hubei sobemo-like virus 41 strain spider133517	99.19	4
45	*Solemoviridae* related	Humaita Tubiacanga virus YN2018	2		Humaita-Tubiacanga virus strain Ab-AAF	99.21	1,572
46	*Solemoviridae* related	Mosquito sobemo-like virus 1*	2	3,055/1,626	Guadeloupe mosquito virus isolate CMS002_019a_SAND	83.45	296
47	*Tombusviridae* related	Culex tombus-like virus 1*	1	3,776	Dansoman virus strain INNplus3391	48.81	385
48	*Tymoviridae* related	Culex quinquefasciatus tymo-like virus 1*	1	7,859	Culex pseudovishnui tymo-like virus 17CxNGK-Cps2-1535	87.01	58
49	*Tymoviridae* related	Guadeloupe Culex tymo-like virus YN2018	1		Guadeloupe Culex tymo-like virus isolate CMS002_022a_SAND	96.74	47
50	*Virgaviridae* related	Aedes binegev-like virus 2 YN2018	2		Aedes binegev-like virus 2 isolate Shenzhen	99.79	387
51	*Virgaviridae* related	Hubei virga-like virus 2 YN2018	1		Hubei virga-like virus 2 isolate CMS001_019_ALCO	98.37	328
52	*Permutotetraviridae* related	Sarawak virus YN2018	1		Sarawak virus strain SWK-M26	97.23	6,826

a Asterisks indicate novel viruses identified in this study.

b RPM, reads per million. The largest one is shown if detected in multiple libraries.

For each library, the number of virus species varied from 2 to16, with the exception of one *C. quinquefasciatus* library from location H, in which no viruses were detected (Fig. 1C). The abundance of each virus varied from 2.00 to 6,825.80 reads mapped per million input reads (RPM) across the pools (Fig. 1D; Table S2). In comparison, the abundance of the mosquito host, determined by *COI* gene sequencing, varied between 1.30 and 774.92 RPM (Fig. 1D; Table S1). A total of 14 viruses, including 2 of *Partitiviridae* (Aedes partiti-like virus 1 [AePLV1], and Aedes partiti-like virus 2), 1 of *Reoviridae* (Aedes reo-like virus 1 [AeRLV1]), 1 *Bunyavirales* (Phasi Charoen-like phasivirus), 2 of *Mononegavirales* (Aedes albopictus anphevirus and Culex quinquefasciatus rhabdo-like virus 1), 2 of *Orthomyxoviridae* (Wuhan Mosquito Virus 6 and Usinis virus), 1 of *Narnaviridae* (Hubei mosquito virus 3), 1 of *Negevirus* (Culex quinquefasciatus negev-like virus 1), 3 of *Solemoviridae* (Guadeloupe mosquito virus, Guangzhou sobemo-like virus, and Humaita Tubiacanga virus), and 1 of *Permutotetraviridae* (Sarawak virus) (Fig. 1D; Table S2), were more abundant than the host *COI* gene (with the highest abundance at 774.92 RPM) (Table S2).

### Phylogenetic relationships and viral genome characterization. (i) Double-stranded RNA viruses.

**FIG 2:**
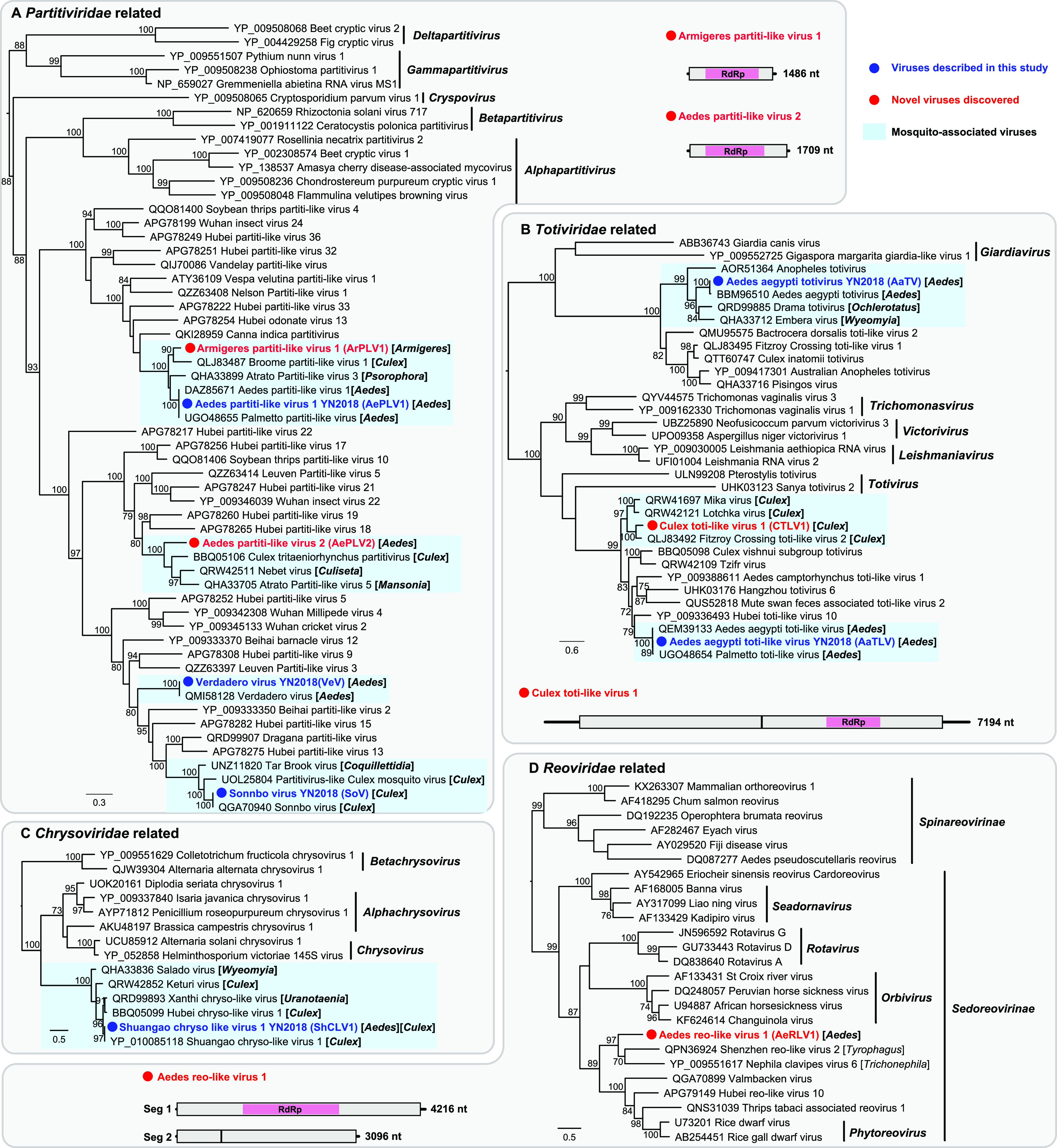
Evolutionary relationships and genomic features of the double-stranded RNA viruses identified in this study. Viruses identified in this study are marked in red/blue and highlighted with a red/blue solid circle. Mosquito-associated viruses are shaded in the blue box. All phylogenetic trees were midpoint rooted for clarity, and only bootstrap values (>70%) are shown adjacent to the nodes. The diagrams provide the genome information of the newly discovered viruses, including the length of each genomic segment, the number of ORFs and the predicted RdRp protein (shown in the pink box).

We identified 10 double-stranded RNA (dsRNA) viruses belonging to *Chrysoviridae* (*n* = 1), *Partitiviridae* (*n* = 5), *Reoviridae* (*n* = 1), and *Totiviridae* (*n* = 3). With the exception of Aedes reo-like virus 1 from *Reoviridae*, the other nine viruses were related to those previously identified in mosquitoes (Fig. 2).

Two novel ArPLV1 and AePLV2 viruses clustered with uncharacterized partiti-like viruses identified from different mosquito genera (Fig. 2A), thereby expanding the known host range of partiti-like viruses. The single-gene segments of both viruses contained a single open reading frame (ORF) sharing 83.47% and 61.17% amino acid similarity with their closest relatives, respectively (Table 1). Like other totiviruses, the Culex toti-like virus 1 (CTLV1) identified in the present study possessed an unsegmented genome comprising two major ORFs (Fig. 2B). CTLV1 clustered with unclassified toti-like viruses also from *Culex* mosquitos and shared 73.20% amino acid similarity with its closest relative (Table 1). Finally, the only novel reovirus identified here, Aedes reo-like virus 1 (AeRLV1), comprised two segments containing three ORFs and was most closely related to Shenzhen reo-like virus 2 from *Tyrophagus* (Fig. 2D). AeRLV1 shared only 30% amino acid similarity over the conserved RdRp region (Table 1) and formed a distant clade with viruses identified from the class *Arachnoidea* (Fig. 2D).

### (ii) Negative-sense, single-stranded RNA viruses.

**FIG 3:**
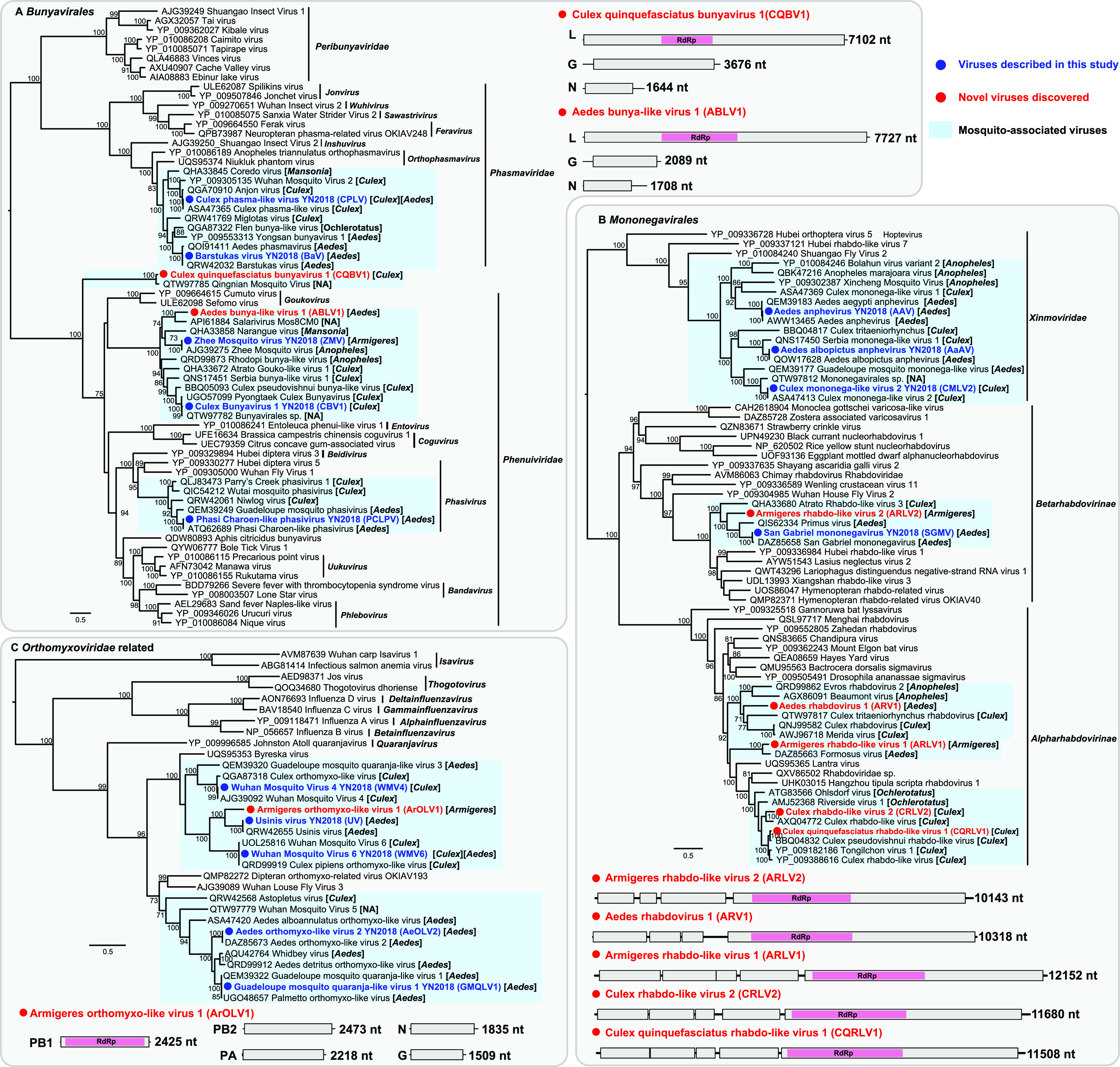
Evolutionary relationships and genomic features of the negative-sense RNA viruses identified in this study. Viruses identified in this study are marked in red/blue and highlighted with a red/blue solid circle. Mosquito-associated viruses are shaded in the blue box. All phylogenetic trees were midpoint rooted for clarity, and only bootstrap values (>70%) are shown adjacent to the nodes. The diagrams provide the genome information of the newly discovered viruses, including the length of each genomic segment, the number of ORFs, and the predicted RdRp protein (shown in the pink box).

Twenty-two negative-sense, single-stranded RNA (–ssRNA) viruses with complete coding regions were identified in this study: 7 fell within the order *Bunyavirales*, 9 fell within the order *Mononegavirales*, and the remaining 6 belonged to the family *Orthomyxoviridae* and a related clade (Fig. 3).

Five of the seven *Bunyavirales* viruses were related to previously described mosquito viruses, with 96 to 100% amino acid identities to their closest relatives, including two *Phasmaviridae* viruses and three *Phenuiviridae* viruses (Fig. 3A). Although Culex quinquefasciatus bunyavirus 1 (CQBV1) fell within the *Bunyavirales* and shared 88.78% amino acid similarity to the most closely related virus (Qingnian mosquito virus [QMV]) (4), both CQBV1 and QMV diverged extremely from previously described viruses and formed a distinct lineage within the *Bunyavirales* (Fig. 3A), exhibiting less than 23% amino acid similarity over the RdRp protein. Similar to the typical genomic structure of the order *Bunyavirales*, the two viruses contained three gene segments. Hence, CQBV1 and QMV may represent a putative new family of the *Bunyavirales* (Fig. 3A). Another novel bunyavirus, Aedes bunya-like virus 1 (ABLV1), identified here also contained three gene segments and fell within the *Phenuiviridae* (Fig. 3A). ABLV1 clustered with reference strains identified in various mosquitoes, exhibiting 48.54% amino acid similarity to the most closely related virus (*Salarivirus*) (Fig. 3A).

We identified five novel viruses clustering within the *Rhabdoviridae*, four of which fell within the subfamily *Alpharhabdovirinae* and one belonging to the *Betarhabdovirinae* (Fig. 3B). Armigeres rhabdo-like virus 2 (ARLV2) was identified in *Armigeres* mosquitos and was closely related to other rhabdoviruses from *Culex* and *Aedes* mosquitoes, sharing low amino acid similarity (less than 53%) with each other. Similarly, Armigeres rhabdo-like virus 1 (ARLV1) and Aedes rhabdovirus 1 (ARV1) clustered with viruses identified from other mosquito species (Fig. 3B), with the largest amino acid similarities of 72.52% and 48.08% over RdRp (Table 1), respectively. Culex rhabdo-like virus 2 (CRLV2) and Culex quinquefasciatus rhabdo-like virus 1 (CQRLV1) were related to Culex rhabdo-like viruses (Fig. 3B). CQRLV1 shared 94.67% amino acid similarity in RdRp to the closest reference sequence—Culex pseudovishnui rhabdo-like virus. Notably, all the five novel *Rhabdoviridae* viruses possessed linear genomes, with typical genome structures comprising 4 to 5 ORFs (Fig. 3B).

One novel Armigeres orthomyxo-like virus 1 (ArOLV1) was identified in *Armigeres* mosquitoes and belonged to an unclassified mosquito-associated clade related to the *Orthomyxoviridae* (Fig. 3C). ArOLV1 clustered with Usinis virus isolated from *Aedes* mosquitoes, with an amino acid similarity of 69.27% in the RdRp protein. Although the number of genome segments in the family *Orthomyxoviridae* ranged from 6 to 8, only 5 genome segments were obtained through sequence similarity from our data, including PB1, PB2, PA, nucleoprotein (N), and the glycoprotein (G) genes (Fig. 3C).

### (iii) Positive-sense, single-stranded RNA viruses.

**FIG 4:**
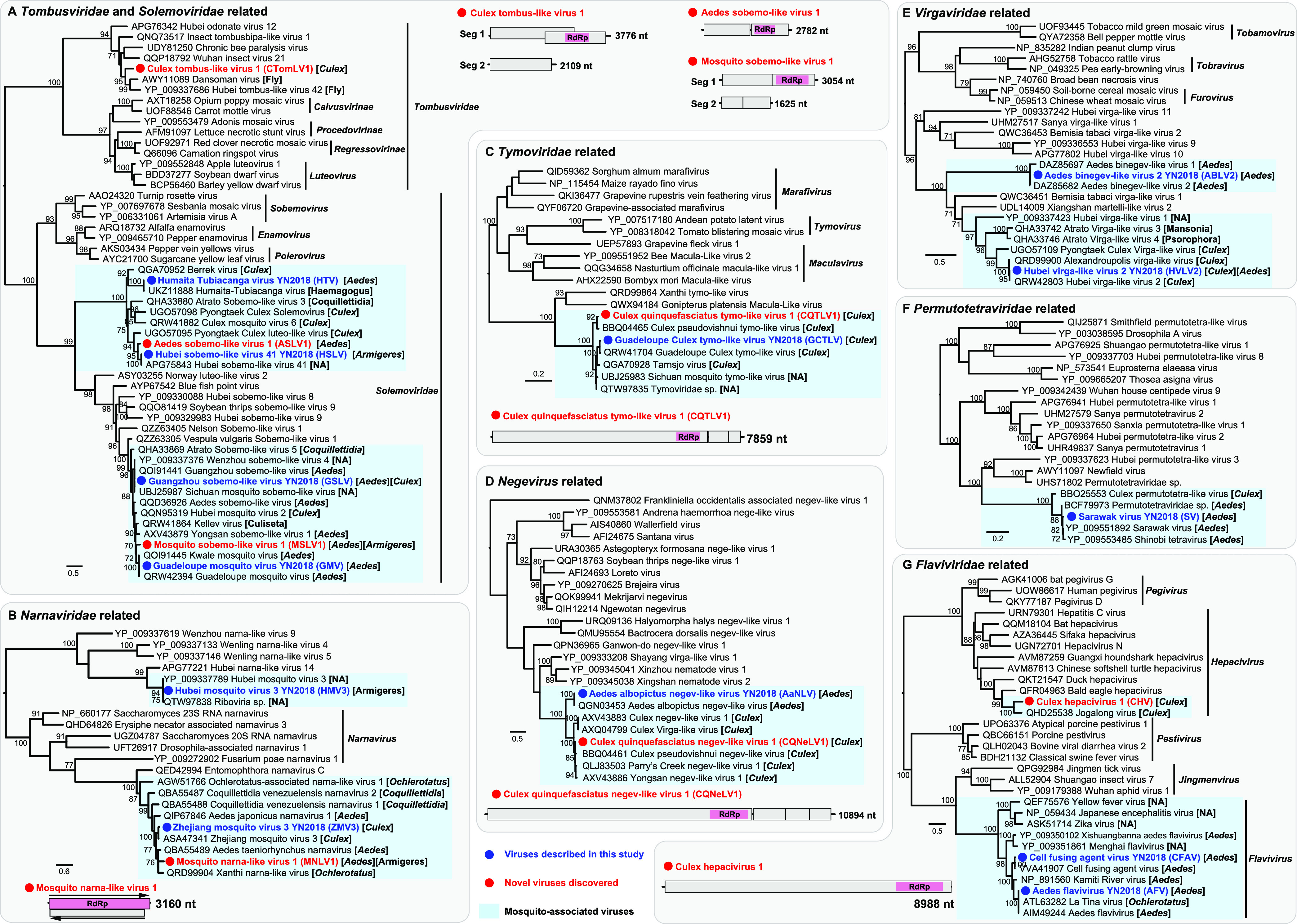
Evolutionary relationships and genomic features of the positive-sense RNA viruses identified in this study. Viruses identified in this study are marked in red/blue and highlighted with a red/blue solid circle. Mosquito-associated viruses are shaded in the blue box. All phylogenetic trees were midpoint rooted for clarity, and only bootstrap values (>70%) are shown adjacent to the nodes. The diagrams provide the genome information of the newly discovered viruses, including the length of each genomic segment, the number of ORFs, and the predicted RdRp protein (shown in the pink box).

A total of 20 positive-sense, single-stranded RNA (+ssRNA) viruses were discovered in this study, grouping within the families *Tombusviridae* (*n* = 1), *Solemoviridae* (*n* = 6), *Tymoviridae* (*n* = 2), *Flaviviridae* (*n* = 3), *Narnaviridae* (*n* = 3), *Virgaviridae* (*n* = 2), *Permutotetraviridae* (*n* = 1), and the clade Negev-like viruses (*n* = 2) (Fig. 4). With the exception of Culex tombus-like virus 1 (CTomLV1) within the *Tombusviridae*, the remaining +ssRNA viruses identified here were most closely related to mosquito-associated viruses (Fig. 4). CTomLV1 was most closely related to Dansoman virus identified in flies, and the viruses exhibited 48.81% amino acid similarity to each other (Table 1). CTomLV1 had the same genomic structure as Dansoman virus, containing two segments: segment 1 carrying two ORFs of a hypothetical protein and RdRp and segment 2 encoding putative capsid protein (Fig. 4A).

We identified two novel viruses of the family *Solemoviridae*: Aedes sobemo-like virus 1 (ASLV1) and Mosquito sobemo-like virus 1 (MSLV1), which were related to Hubei sobemo-like virus 41, previously identified in mosquitoes from China, and Guadeloupe mosquito virus from the Caribbean, respectively (Fig. 4A). ASLV1 and MSLV1 had similar genome structures to their closest relatives and exhibited RdRp amino acid identities of 87.6% and 83.45%, respectively (Table 1). Notably, MSLV1 was identified in both *Aedes* and *Armigeres* mosquitoes (Fig. 4A), showing broader host ranges (host-sharing events) across different mosquito genera.

Another host-sharing event was identified in the novel Mosquito narna-like virus 1 (MNLV1), which was also found in both *Aedes* and *Armigeres* mosquitoes (Fig. 4B). MNLV1 was closely related to reference strains identified in *Aedes*, *Culex*, *Coquillettidia*, and *Ochlerotatus* mosquitoes and shared 36.17% amino acid similarity with Ochlerotatus-associated narna-like virus 2. MNLV1 had the same genome structure as its closest relative, with a dual-coding genome structure: two ORFs cover both the sense and antisense genomes, encoding RdRp and a hypothetical protein (Fig. 4B).

We also identified two novel viruses related to the *Tymoviridae* and Negev-like viruses (Fig. 4C and D). Culex quinquefasciatus tymo-like virus 1 (CQTLV1) clustered within the clade of mosquito-associated viruses identified in *Culex* mosquitoes (Fig. 4C), sharing 87.01% amino acid similarity with Culex pseudovishnui tymo-like virus. Similarly, Culex quinquefasciatus negev-like virus 1 (CQNeLV1) and Culex mosquito-associated negev-like viruses formed a clade and showed 90.05% amino acid similarity with Parry’s Creek negev-like virus 1 (Fig. 4D).

Of particular note was the identification of a novel hepacivirus, termed Culex hepacivirus 1 (CHV1), from a Culex quinquefasciatus mosquito library (Fig. 4G). CHV1 was related to a previously described mosquito-associated virus (Jogalong virus [JgV]) (16), which was identified in Culex annulirostris mosquitoes from Western Australia, sharing 54.62% amino acid sequence similarity with each other. Notably, CHV1 and JgV clustered within the clade of hepaciviruses associated with avian hosts (Fig. 4G), sharing less than 43% amino acid similarity with its closest relative, Bald eagle hepacivirus (Fig. 4G).

### Factors affecting the structure and abundance of mosquito viromes.

**FIG 5:**
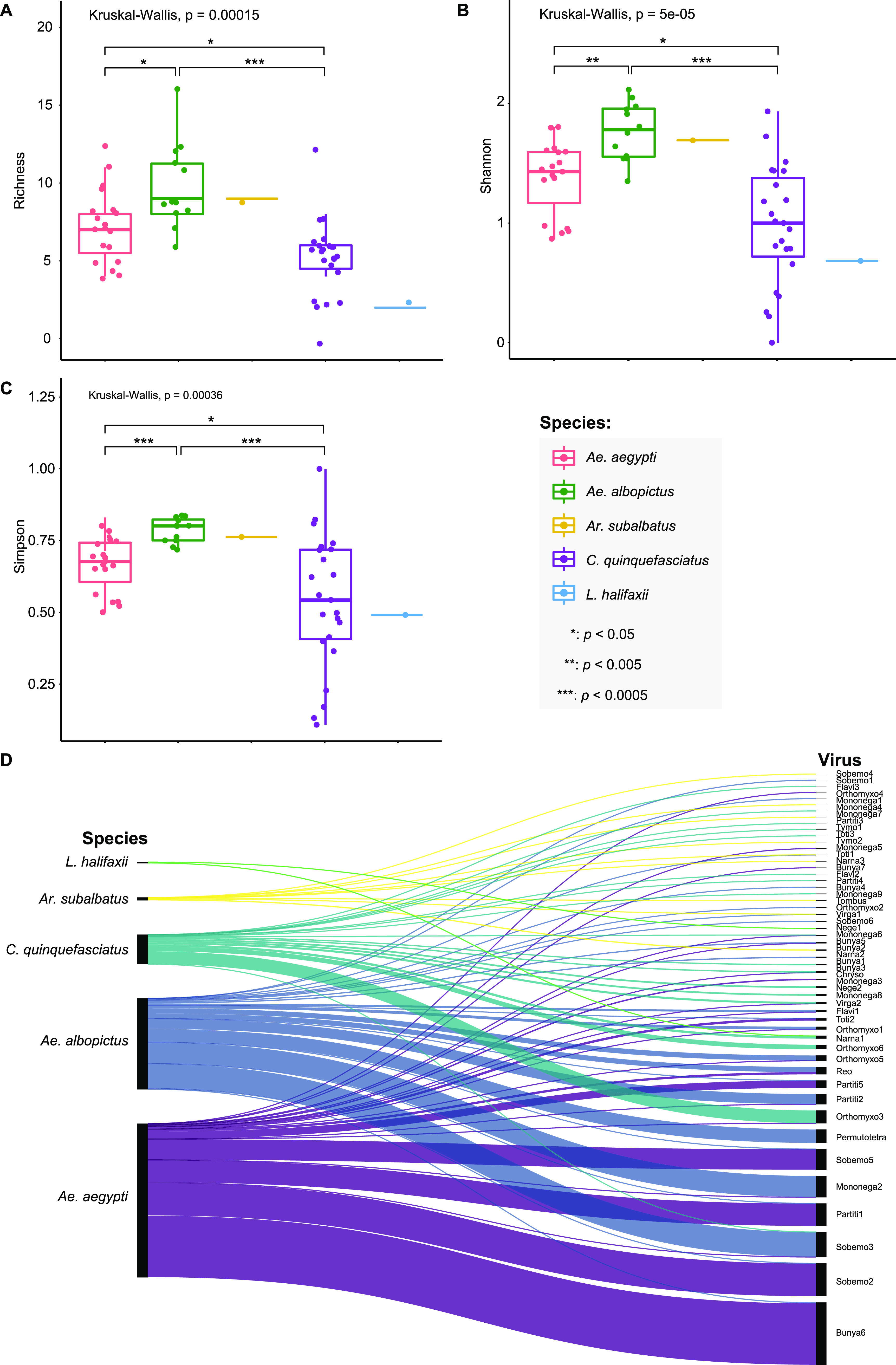
Comparison of the viral diversity between mosquito species. (A) Virome richness, (B) Shannon index, and (C) Simpson index showing the differences of virome composition between mosquito species; (D) Sankey diagram of the virome compositions of different mosquito species. The thickness of links in the Sankey diagram is proportional to the total abundance (as measured by RPM) of each virus across the samples studied.

Virus compositions and abundances differed substantially between mosquito species. In general, *Aedes* mosquitoes (both *Ae. aegypti* and *Ae. albopictus*) contained more viruses than *Culex* mosquitoes (Fig. 1C) and also had higher abundance (Fig. 1D). The highest viral richness was found in *Ae. albopictus* mosquitoes, with a median of 9.0 (Fig. 5A), followed by *Ae. aegypti* and *C. quinquefasciatus* mosquitoes, with medians of 7 and 6, respectively (Fig. 5A). Similarly, both the Shannon (Fig. 5B) and Simpson (Fig. 5C) effective indices were the highest in *Ae. albopictus*, followed by *Ae. aegypti*, and the lowest values of the two indices were from *C. quinquefasciatus* (Fig. 5).

**FIG 6:**
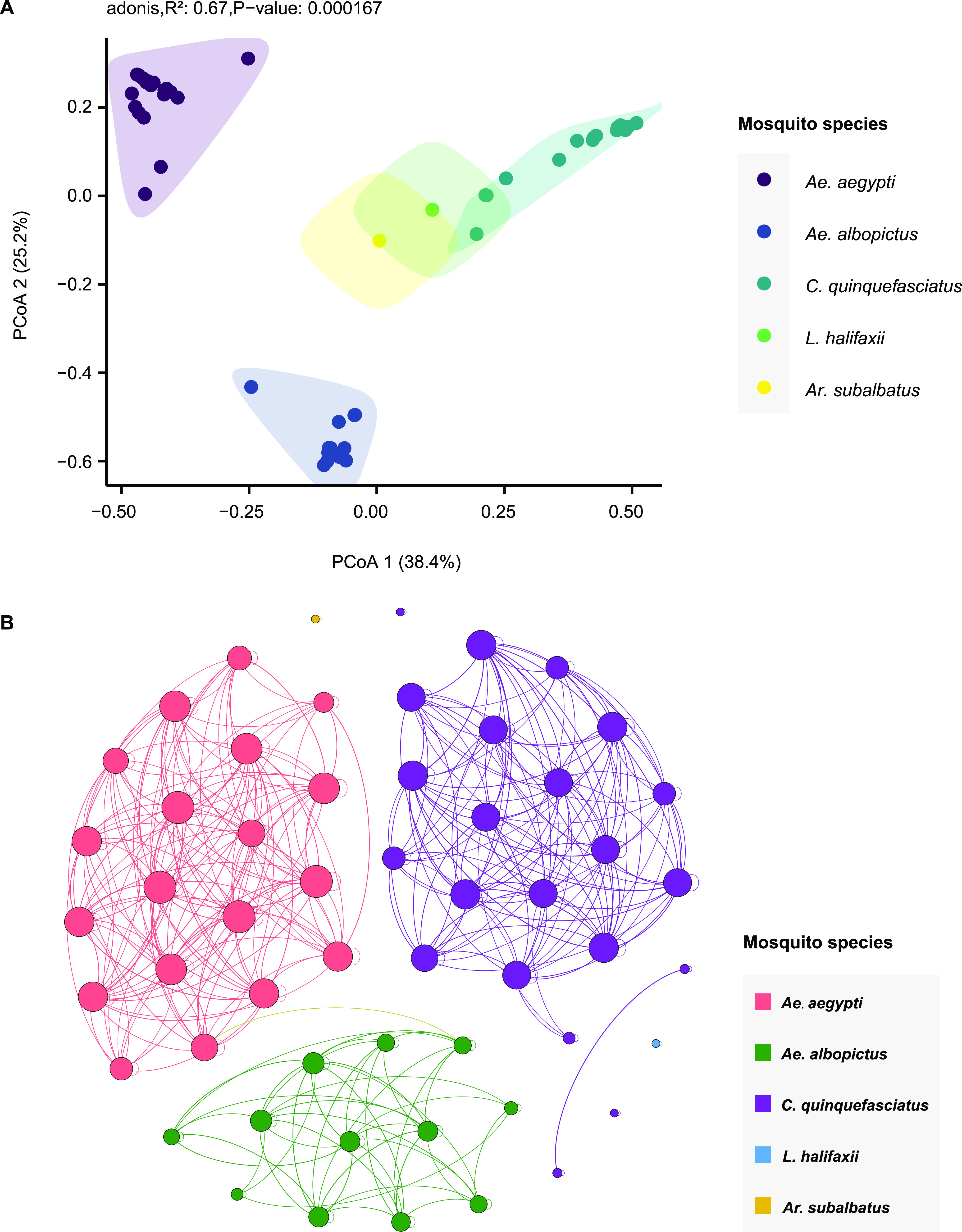
β-diversity analysis and viral co-occurrence network. (A) β-diversity analysis of the virome composition of different mosquito species; (B) viral co-occurrence network of host taxa. The network was calculated using the relative abundance of each virus across libraries. Each node represents a library. Libraries with a Spearman’s correlation greater than 0.6 (*P* < 0.05) are connected by edges. The thickness of each edge is determined according to the correlation coefficient; the size of a node is determined by the degree (number of edge connections). The nodes were colored by mosquito species.

Of the 52 viral species discovered, 5 were shared between *Aedes* and *Culex* mosquitoes (Fig. 5D; Table S2), and only 2 were shared between *Aedes* and *Armigeres* species (Fig. 5D). No viruses were shared between *Culex* and *Armigeres* mosquitoes (Fig. 5D). Notably, *Ae. aegypti* shared 14 viruses with *Ae. albopictus*, while *C. quinquefasciatus* shared one virus with other *Culex* species (Lutzia halifaxii), although only two viruses were discovered in *L. halifaxii* (Fig. 5D). The clustering of the libraries with similar viromic composition and abundance was further described using β-diversity analysis. Principal-coordinate analysis (PCoA) plots based on the Bray-Curtis distance matrices revealed that samples from the same mosquito species clustered together (adonis; *R*^2^ = 0.67, *P* = 0.0002) (Fig. 6A), demonstrating that mosquito species affect virome structure.

The possible association between sampling locations and viral composition and abundance was examined further (Fig. S1) by measuring observed richness (Fig. S1A), the Shannon index (Fig. S1B), and the Simpson index (Fig. S1C). Three locations (D, E, and F) contained only one or two libraries with limited virome diversity. Only location H showed a significant difference from locations B and C, suggesting a potential association between the geographic location and the viromic structure of mosquitoes. In addition, PCoA plots based on the Bray-Curtis distance matrices revealed that samples from different locations did not form distinct clusters (Fig. S1D). Hence, there is no strong evidence for geographic structure in mosquito viromes in this study, and the mosquito collection sites were close to each other in this study.

We further constructed a co-occurrence network among mosquito species based on significant positive correlations (Spearman’s ρ > 0.6; *P* < 0.05) (Fig. 6B). The network was derived from the relative abundance of each virus, comprising 56 nodes (56 mosquito libraries) and 372 edges. Based on the modularity class, the entire network could be parsed into three major modules, corresponding to the three major mosquito species (Fig. 6B). Notably, nodes were inclined to interact more with nodes within the same module than with nodes of other modules. The co-occurrence patterns clearly illustrated the correlations between the mosquito species and viral abundance; however, the network among sampling locations showed no obvious correlations (Fig. S2).

### *Wolbachia* diversity and abundance.

**FIG 7:**
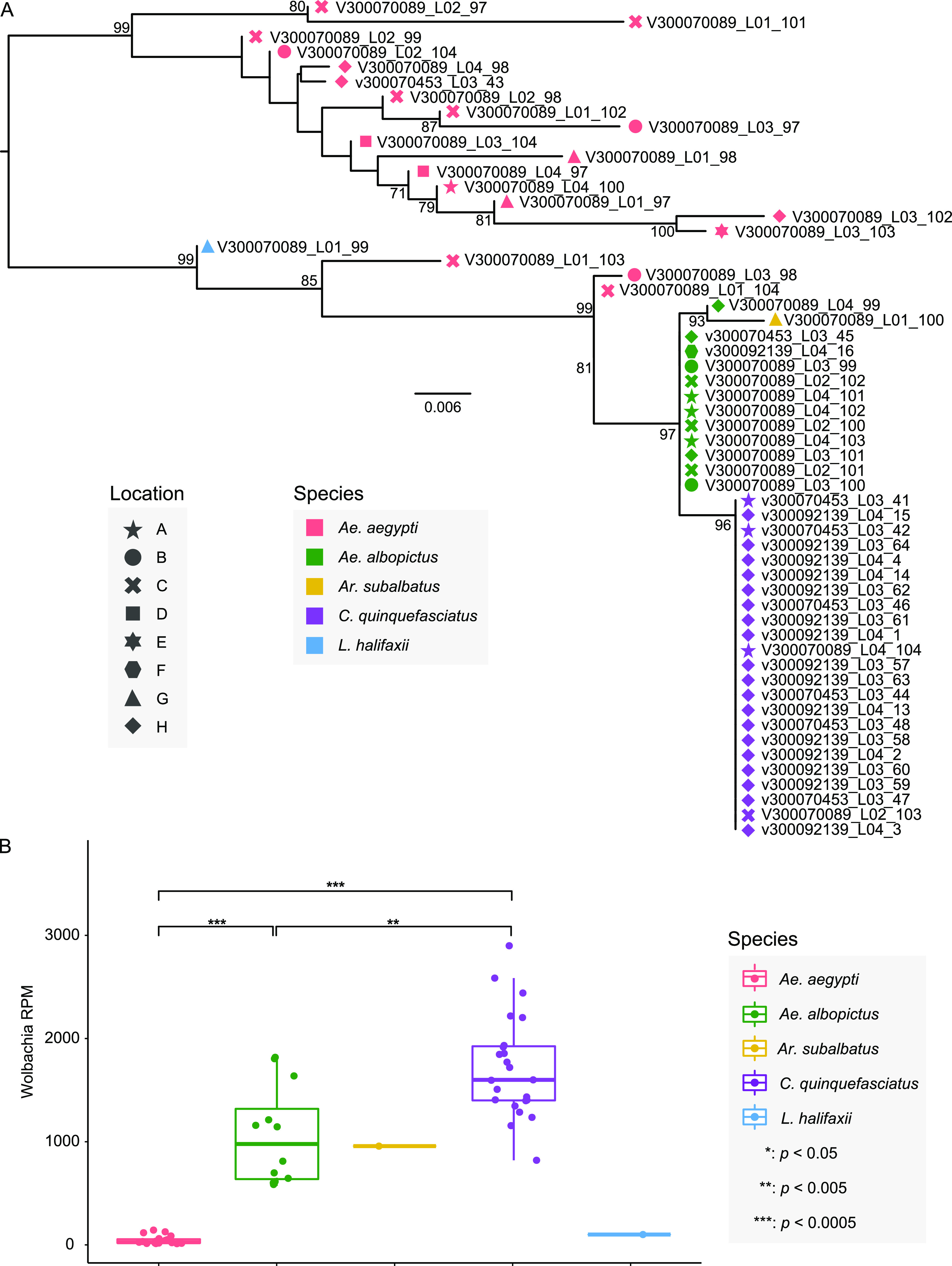
Genetic diversity and abundance of the *Wolbachia* bacteria. (A) Phylogenetic analysis of the *Wolbachia* bacteria identified in this study performed using the 16S rRNA gene sequences assembled from the sequencing data. Different shapes and colors indicate the sampling location and mosquito species, respectively. (B) Comparison of abundance of *Wolbachia* across five mosquito species.

As an endosymbiont bacterium, *Wolbachia* was detected in all libraries in this study, with relatively high abundance (11.72 to 2,899.50 RPM) (Fig. 1D; Table S2). The alignment of *Wolbachia* 16S rRNA sequences showed 87.99 to 100% nucleotide identities to each other, and phylogenetic analyses revealed two major lineages (Fig. 7A): the first comprised all *Wolbachia* sequences obtained from *Ae. aegypti*, while the other contained *Wolbachia* sequences from all five mosquito species. The phylogenetic tree indicated that *Wolbachia* sequences from *Ae. aegypti* had higher genetic diversity, while *Wolbachia* sequences from *Ae. albopictus* and *C. quinquefasciatus* were more genetically homogeneous. Further comparisons of the abundance (RPM) of *Wolbachia* gene sequences across libraries revealed significant differences between *Ae. aegypti*, *Ae. albopictus*, and *C. quinquefasciatus* (Fig. 7B). The highest abundance of *Wolbachia* sequences was observed in *C. quinquefasciatus*, with a median of 1,599.5 RPM, followed by *Ae. albopictus* mosquitoes (median of 977.8), and the lowest abundance was in *Ae. aegypti* mosquitoes, with a median of 25.5 (Fig. 7B).

## DISCUSSION

We present a comprehensive description of the viromes of 991 mosquitoes collected from eight locations in Yunnan Province in southwestern China. Although previous metagenomic studies have revealed numerous novel and highly divergent RNA viruses in mosquitoes, analysis of the transcriptomes of the five mosquito species in the present study has led to the discovery of 52 RNA viruses belonging to 15 viral families and unclassified clades. Notably, 19 viruses were novel, with half sharing 30 to 70% amino acid similarity to their most closely related viruses. One of the most notable discoveries was Culex quinquefasciatus bunyavirus 1 (CQBV1), which represented a putative new virus family within the order *Bunyavirales* together with previously described QMV (4). These results underscore the capacity of mosquitoes to harbor a wide diversity of RNA viruses, highlighting the necessity for constant surveillance of potential viral pathogens in these arthropod vectors.

Another key result from our study was that within the *Aedes* mosquito vector species, both *Ae. aegypti* and *Ae. albopictus* harbored significantly greater virus diversity than *Culex* mosquitoes (Culex quinquefasciatus). This is in contrast to the observation in a previous report that *Culex* species harbored more viruses at high abundance than *Aedes* mosquitoes (17). The underlying explanation for these contrary results could be due to uneven sample sizes of each mosquito genus. Our results also revealed pronounced differences between the virome structures of *Ae. aegypti* and *Ae. albopictus* mosquitoes: even though there is considerable overlap in the viruses carried by these two *Aedes* mosquitoes, the latter had both higher diversity and viral abundance. These findings were also supported by further statistical and multidimensional scaling analyses and are consistent with prior evidence that different *Aedes* mosquitoes can have significantly different virome compositions (17, 18). Hence, the ecology of mosquito viruses was driven, at least in part, by host taxon, consistent with the predicted narrow host range of insect-specific viruses (6).

In contrast, according to the α- and β-diversity analyses, the virome structures were relatively homogeneous across different locations. Indeed, some mosquito-specific viruses exhibiting high similarity have been described from different continents (19), indicating that viruses can be transmitted to wide geographical areas through mosquito populations. As all of the sampling sites in the present study were geographically close, larger-scale samplings covering different ecological niches are clearly required for further investigations with respect to the correlation between geographic location and virome structure.

The viruses discovered here expand the host ranges of several mosquito viruses to include additional mosquito species and even genera. For example, Shuangao chryso-like virus 1 YN2018 (ShCLV1), Culex phasma-like virus YN2018 (CPLV), and Guangzhou sobemo-like virus YN2018 (GSLV) were identified in both *Aedes* and *Culex* mosquitoes, while Mosquito narna-like virus 1 (MNLV1) was present in both *Aedes* and *Armigeres* mosquitoes with high abundance, suggesting host sharing and the intergeneric transmission of these viruses. However, those viruses that clustered with viruses associated with fungi rather than mosquitoes or arthropods might have been derived from other eukaryotic organisms present in the mosquito microbiome or from fungal infections of the mosquito cuticle.

According to the WHO (20), the most prevalent viral infections are primarily transmitted by *Ae. aegypti*, *Ae. albopictus*, and *C. quinquefasciatus*, including Zika virus fever, dengue, yellow fever, Japanese encephalitis, and West Nile fever. However, surprisingly, none of these known viral pathogens were identified in this study. Interestingly, one virus, CHV, as well as the previously documented JgV (16), clustered with vertebrate-associated hepaciviruses. Phylogenetic analyses of the *Flaviviridae* suggested that both CHV and JgV were likely associated with avian hosts, rather than the mosquito itself. Indeed, through targeting two of the vertebrate mitochondrial genes (*COI* and *Cytb*), JgV was suspected as a contamination from a blood meal taken from a bird host (16). However, we did not find vertebrate mitochondrial genes from the sequencing data. Given the high divergence of these mosquito hepaciviruses, it will be important to investigate their true natural host, particularly as this may provide valuable information on their evolutionary history. Although analysis of host genes from mosquito sequencing data is highly suggestive of the natural host, viruses detected in the blood of vertebrate species would provide convincing evidence of the real source of the viruses. To this end, more expansive surveillance is required, including a larger collection of mosquitoes and blood samples from vertebrate animals present in the same location.

*Wolbachia* has been documented to provide resistance to the infection with some viruses, such as dengue virus and Zika virus in mosquitoes (21, 22), and hence has been suggested as a potential tool for vector-borne disease control. However, not all *Wolbachia* strains have clear effects in inhibiting virus replication, and *Wolbachia* infection does not protect against all viruses (23, 24). Despite this, little is known about how the ecology of hosts impacts *Wolbachia* diversity. We found *Wolbachia* in all libraries, once again indicating the prevalence of *Wolbachia* in mosquitoes in China (25). However, there was a large discrepancy in the genetic diversity and abundance of *Wolbachia* between different mosquito populations. Specifically, *Wolbachia* in *Ae. aegypti* had high diversity but low abundance, while the converse was seen (low diversity/high abundance) in both *Ae. albopictus* and *C. quinquefasciatus*. These results suggested that mosquito species might also play an important role in *Wolbachia* composition.

We do not believe that the *Wolbachia* sequences identified in *Ae. Aegypti* mosquitoes result from contamination as the *Ae. aegypti* libraries were sequenced on different lanes and sequences within the same lane did not share 100% nucleotide identity. In addition, *Wolbachia*-infected *Ae. Albopictus* mosquitoes, not *Ae. Aegypti*, are being released in a few small independent islands in China (26), and our samples were not collected from these sites. However, *Ae. aegypti* was not thought to naturally harbor *Wolbachia* (27) until recently in some Southeast Asian countries (such as Malaysia, India, the Philippines, and Thailand) as well as the United States (28, 29). None of these studies provide robust evidence that *Ae. aegypti* harbors natural *Wolbachia* infections. The presence of natural *Wolbachia* infections may interfere with compatibility patterns between *Ae. aegypti* mosquitoes and some *Wolbachia* strains (27). Confirmation of a natural infection in these mosquitoes will require significant additional experimental work.

In conclusion, we studied the viral diversity of five mosquito species sampled in different locations in Yunnan Province and highlighted the capacity of mosquitoes to harbor a rich diversity of RNA viruses. The viral compositions varied mainly between different mosquitoes, suggesting host species represents an important factor shaping the virome composition of mosquitoes.

## MATERIALS AND METHODS

### Sample collection.

From October to December 2018, a total of 991 adult mosquitoes were collected using light-traps from eight locations in Jinghong City, Xishuangbanna, Yunnan Province, China (Fig. 1). Mosquito species were initially identified morphologically by experienced field biologists and further confirmed based on sequences of the cytochrome *c* oxidase subunit 1 mitochondrial gene (*COI*). All samples were divided into 56 pools by mosquito species and geographic location and were transported to the laboratory on dry ice.

### Metatranscriptomic sequencing.

Mosquitoes were rinsed three times using RNA- and DNA-free phosphate-buffered saline (PBS) solution (Gibco) before homogenization with steel beads in PBS solution. Total RNA was extracted using TRIzol reagent (Invitrogen). RNA quantity and quality were checked using the Agilent 2100 Bioanalyzer (Agilent). Host rRNA was removed using the MGIEasy rRNA depletion kit according to the manufacturer's instructions, and sequencing libraries were constructed using the MGIEasy mRNA library prep kit. Paired-end (100-bp) sequencing of each RNA library was performed on the BGISEQ-500RS sequencing platform (BGI).

### Data analysis and virus discovery.

A quality assessment of the raw sequencing reads was conducted using the Fastp v.0.19. (30) and Trimmomatic (31) programs, before *de novo* assembly using the Trinity program (32). The assembled contigs were then compared against the nonredundant nucleotide (nt) and protein (nr) databases downloaded from NCBI using blastn (33) and Diamond blastx (34), with cut-off E values of 1 × 10^−10^ and 1 × 10^−5^, respectively. All potential viral contigs were identified and merged into longer viral contigs using Geneious Prime (35). False-positive results due to cross-contamination and index hopping during sequencing were excluded as previously described (36). The relative abundance of the viruses identified was determined by mapping the reads back to the assembled contigs using Bowtie2 v.2.3.3.1 (37) and calculated as the number of reads mapped per million input reads (RPM) using the formula “total mapped reads/total reads × 1 million.” Bowtie2 was used to align the reads to each novel virus genome, and SAMtools (38) was used to compute the percentage of reads mapped and coverage depth. Novel viruses were defined employing the previously defined criterion such that the translated protein sequence shared less than 90% amino acid similarity in the RNA-dependent RNA polymerase (RdRp) to any previously described viruses (39).

### Phylogenetic analyses.

RdRp sequences of the viruses identified from this study were then aligned with their corresponding homologs in reference viruses using the MAFFT v.7.407 program (40) employing the E-INS-I algorithm, followed by the removal of ambiguously aligned regions using TrimAl v.1.4 (41). Phylogenetic trees were constructed using the maximum likelihood method implemented in IQ-TREE v.1.6.12 (42), employing the best-fit substitution models identified by IQ-TREE.

### Ecological dynamics analysis and network analysis.

Statistical analyses of viral genetic diversity and abundance were performed using the *t* test or Wilcoxon test based on the results of a normal distribution test (Shapiro-Wilk test) in the ggpubr package and were plotted using the ggplot2 package in RStudio v.4.1.2. The observed richness, Shannon index, and Simpson index (i.e., α diversity) were estimated for each library using modified Rhea script sets (43) and compared between different mosquitoes using the Kruskal-Wallis rank sum test. Principal-coordinate analysis (PCoA) (i.e., β diversity) was performed based on the Bray-Curtis dissimilarity matrix using the Vegan package (44). A correlation between two items was considered statistically robust if the Spearman's correlation coefficient (ρ) was >0.6 and the *P* value was <0.05, with the *P* value adjusted with a multiple-testing correction using the Benjamini-Hochberg method (45). All pairwise Spearman's rank correlations between the viral members were calculated using the psych package in RStudio v.4.1.2. Network visualization was conducted on the interactive platform of Gephi (46).

### Identification of *Wolbachia* bacteria.

Bacteria were initially identified in the metatranscriptomic data using MetaPhlAn2 (47). The 16S rRNA gene was used to conduct phylogenetic analyses and similarity comparisons for *Wolbachia*. To estimate their relative abundance, sequence reads were mapped to the complete reference genomes (CP031221 [Wolbachia pipientis wAlbB chromosome]) from which the RPM was calculated.

### Ethics statement.

This study was performed in accordance with the institutional and national guidelines for the care and handling of the animals.

### Data availability.

All sequence reads generated in this study have been uploaded into the NCBI Sequence Read Achieve (SRA) database under BioProject accession no. PRJNA911492. All novel and known virus genome sequences generated in this study have been deposited in NCBI/GenBank under accession no. OQ067620 to OQ067711.
