# Supplementary material for: Metatranscriptomic Sequencing Reveals Host Species as an Important Factor Shaping the Mosquito Virome
Source: Microbiol Spectr. 2023 Feb 14;11(2):e04655-22. doi: 10.1128/spectrum.04655-22 (PMC10101097; doi:10.1128/spectrum.04655-22)
Supplement: Supplemental file 1 — Fig. S1 and S2. Download spectrum.04655-22-s0001.pdf, PDF file, 1.1 MB [file spectrum.04655-22-s0001.pdf]

# Supplemental Figures

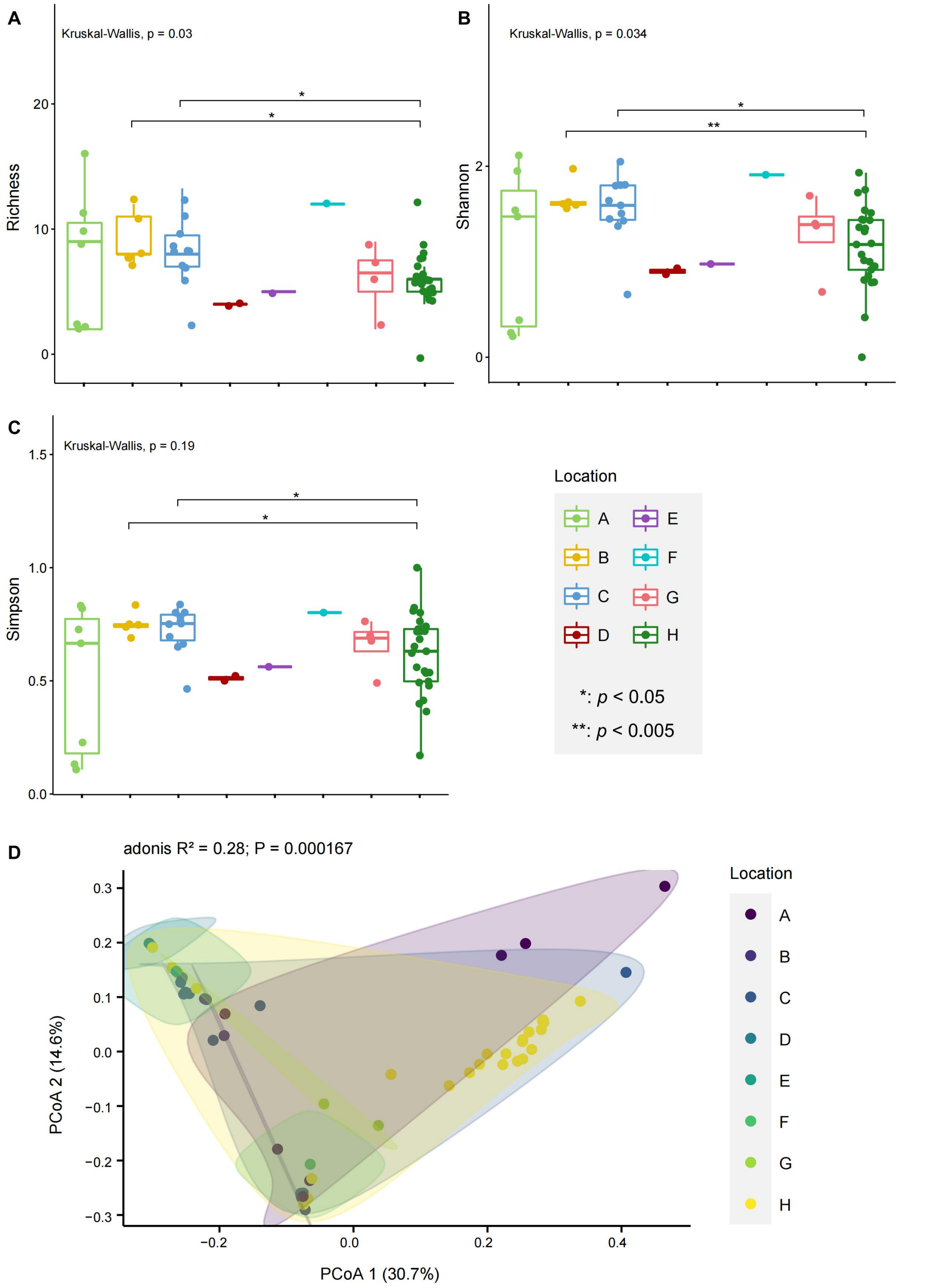


**Figure S1. Comparison of the viral diversity across different sampling locations.** (A) Virome richness, (B) Shannon index, (C) Simpson index, and (D) Beta diversity analysis of the viromic composition across different sampling locations.

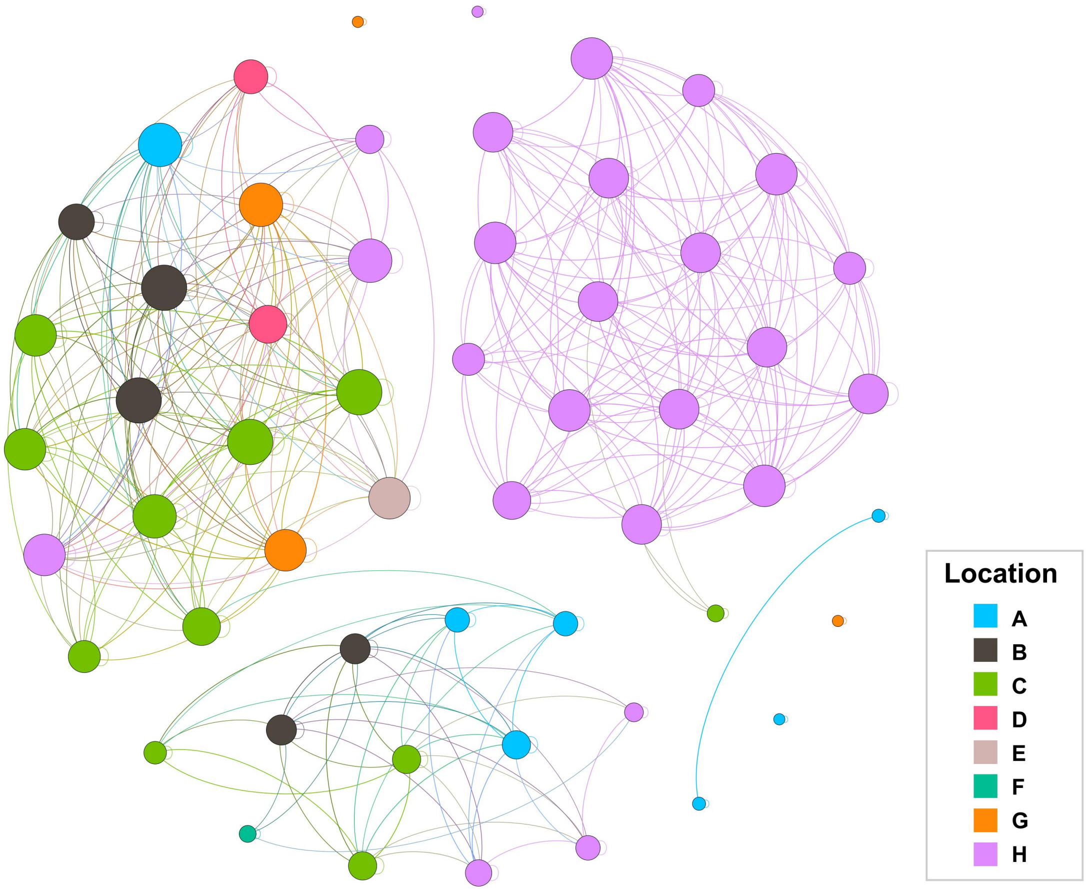


**Figure S2. Viral co-occurrence network of geographical locations.** The network was calculated using the relative abundance of each virus across libraries. Each node represents a library. Libraries with a Spearman's correlation greater than 0.6 ($P < 0.05$) are connected by edges. The thickness of each edge is determined according to the correlation coefficients; the size of a node is determined by the degree (number of edge connections). The nodes were colored by sampling locations.
